# Surveillance of Parenting Outcomes, Mental Health and Social Support for Primiparous Women among the Rural-to-Urban Floating Population

**DOI:** 10.3390/healthcare9111516

**Published:** 2021-11-06

**Authors:** Jiemin Zhu, Ziwen Ye, Qiyu Fang, Lingling Huang, Xujuan Zheng

**Affiliations:** 1 Department of Nursing, School of Medicine, Xiamen University, Xiamen 361000, China; Jieminzhu@xmu.edu.cn; 2 Health Science Centre, School of Nursing, Shenzhen University, Shenzhen 518060, China; y673817550@126.com (Z.Y.); fangqiyu320@szu.edu.cn (Q.F.); huanglingling@szu.edu.cn (L.H.)

**Keywords:** postpartum women, floating women, parenting, self-efficacy, postpartum depression, social support, mainland China

## Abstract

China has the largest population of floating rural-to-urban women worldwide, most of whom are of childbearing age. However, few studies have been conducted to monitor the changing trends in parenting outcomes, mental health and social support for these women in the early postpartum period. In this quantitative longitudinal study, 680 primiparous women among the floating population were recruited in Shenzhen, China. Face-to-face collection of socio-demographic questionnaires was completed by researchers in maternity wards on the third postnatal day. Follow-up electronic questionnaires were dispatched to women via email or WeChat at 6 weeks and 12 weeks following childbirth, including the Self-efficacy in Infant Care Scale (SICS), Edinburgh Postnatal Depression Scale (EPDS) and Postnatal Social Support Scale (PSSS), to measure maternal self-efficacy (MSE), postpartum depression (PPD) and social support, respectively. The mean scores of MSE for these floating women were 67.16 (14.35) at 6 weeks postpartum and slightly increased to 68.71 (15.00) at 12 weeks postpartum. The mean scores of EPDS remained almost stable, from 11.19 (4.89) to 11.18 (5.34) at the two time points. The prevalence of mild and severe PPD among floating women at 6 and 12 weeks after childbirth decreased from 54.4% to 40.1% and from 50.6% to 35.4%, respectively. The mean score of social support was 37.04 (10.15) at 6 weeks postpartum and slightly improved to 38.68 (10.46) at 12 weeks postpartum. Primiparous women among the rural-to-urban migrant population had an obviously negative status of parenting outcomes and mental health; and there was a lack of social support after childbirth. In future, tailored evidence-based interventions are highly needed to promote floating women’s parenting outcomes, mental wellbeing and social support in the early stages of motherhood. As a higher-risk group of PPD, primiparous women among the floating population require effective and accessible mental health care after childbirth, such as early PPD screening and timely therapeutic methods.

## 1. Introduction

Due to rapid urbanization processes, China has witnessed a great number of people migrating from rural to urban areas during the past 30 years, called a “floating population” or “internal migrants” [1,2]. In 2020, the floating population in China was about 247 million and accounted for approximately 18% of the total population. One survey reported that almost 50% of migrant people are women, and the overwhelming majority of these women are of reproductive age [3,4,5]. Research has found that imbalance in the allocation of health resources has led to health inequalities between the city residents and floating women [6,7]. For example, compared with city residents, floating women of childbearing age are likely to suffer from more reproductive problems and use fewer perinatal health services, such as antenatal care and postnatal home visiting [5,8]. Therefore, the wellbeing of rural-to-urban floating women of reproductive age ought to attract significant attention from the government, health professionals and researchers [1,8,9].

The motherhood transition poses tremendous challenges for primiparous women to acquire various parenting knowledge and skills, adapt to changing family relationships, and accept the role of a mother [10]. Due to a lack of parenting experience, many first-time mothers find it too hard to cope with these physical, mental and social challenges during the initial postpartum stage [11,12]. Researchers have found that these new mothers frequently encountered inefficient mother–infant interactions and various unsuccessful parenting tasks, which negatively affected the physical and mental wellbeing of mothers and infants [13,14].

Maternal self-efficacy (MSE) refers to the beliefs that women hold about their capability to organize and perform different parenting tasks [15]. Sound evidence has identified that MSE is not only the significant indicator of parenting outcomes, but is related to the wellbeing of women and children [13,14]. Considering the important impacts of MSE, related studies have been conducted in various countries worldwide [16,17,18,19]. However, the majority of research has focused on well-educated and employed city women [13,20], and little research is related to rural-to-urban floating women, who exhibit characteristics such as poorer educational qualifications, economically strained conditions, and worse working status [9].

The two main factors influencing MSE have consistently been identified as postpartum depression and social support [13,20,21]. Postpartum depression (PPD) or postnatal depression (PND), with a high incidence rate and severe harm to the mother, infant and family, has become a serious global public health problem [22]. The prevalence of PPD was reported to be 19.8% postnatally in low- and middle-income countries [23]. Study findings showed that PPD affects 15–30% of Chinese mothers after delivery [24]. The significantly negative association between PPD and MSE was verified, and mothers exhibiting fewer PPD symptoms were prone to having higher MSE levels [17,21,25,26].

The other significant factor impacting on women’s MSE is that of social support [13,20,21], including the perception of available support and the satisfaction with received assistance [27]. This may be divided into structural and functional modules [28]. Structural social support is regarded as a formal social network (health professionals) and an informal social network (family members and friends). Functional social support refers to informational, instrumental, emotional and appraisal support [28]. Research findings have highlighted that social support positively affects MSE, and women receiving more social support after delivery were prone to higher MSE levels [18,21,26,29,30].

In mainland China, studies relating to MSE have been limited to first-time mothers among local city residents [13,14], and little research relating to MSE has been conducted for rural-to-urban migrant women [9]. In particular, the changing trends in parenting outcome, mental health and social support were not clear for these women after childbirth. Therefore, this research aimed to monitor the changing trends in MSE, PPD and social support for new mothers among floating women in the early postpartum stage.

## 2. Materials and Methods

### 2.1. Participants

This study was conducted in the maternity wards of two public tertiary hospitals in Shenzhen City, China, between 2019 and 2020, where more than 4000 live births were recorded per year in each study hospital. Information sheets were distributed to all eligible women to introduce the purposes and process of research before recruitment. Rural-to-urban floating women were recruited according to the inclusion and exclusion criteria (presented in Table 1) [9]. The participants were informed of their freedom to withdraw at any time and were assured of anonymity through the use of special code numbers to identify themselves. Informed consent was obtained from every participant before data collection.

### 2.2. Procedure

**Figure 1:**
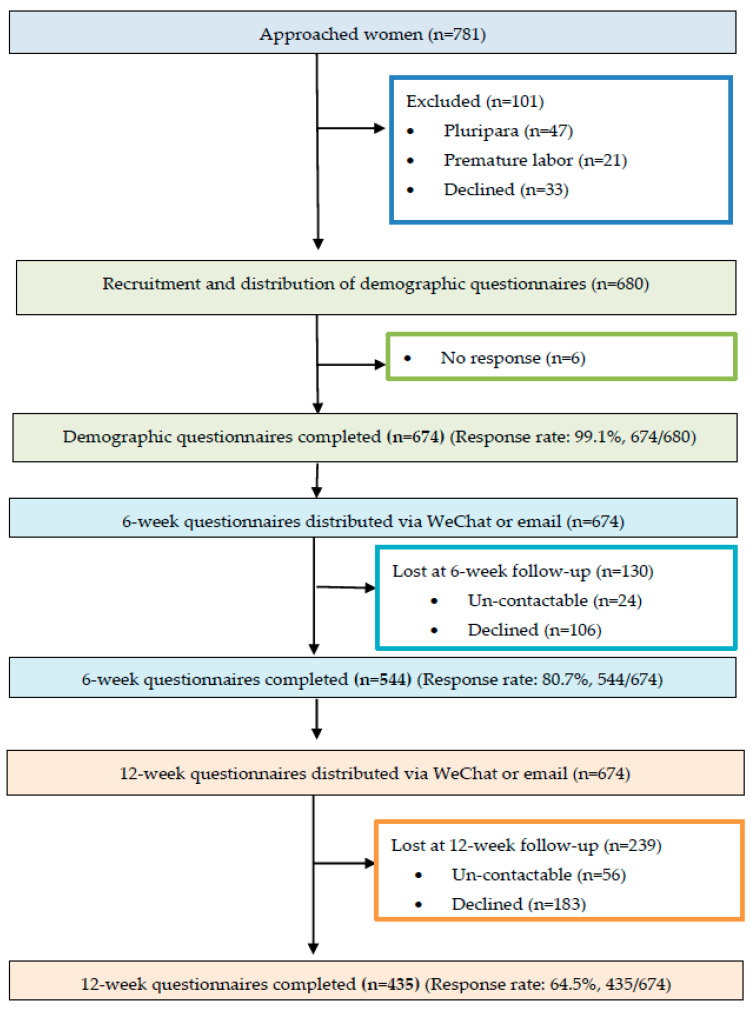
Flow diagram of participants and data collection.

On the third postnatal day, the research team collected the contact details of participants and their completed socio-demographic questionnaires in the maternity wards of two public tertiary hospitals. The follow-up electronic questionnaires comprising SICS, EPDS and PSSS were dispatched to women via email or WeChat at 6 and 12 weeks postpartum. A total of 544 participants completed 6-week questionnaires, and 435 completed 12-week questionnaires, which were returned to the research team likewise via WeChat or email. A flow diagram of participant and data collection is shown in Figure 1. In order to increase the response rate, primiparous women were given friendly WeChat reminders one week and one day before the 6-week and 12-week postpartum points. All collected data were treated confidentially and anonymously.

### 2.3. Measures

#### 2.3.1. Socio-Demographic Questionnaire

A socio-demographic questionnaire was developed to collect the social–demographic information of childbearing age, marital status, monthly family income, education level, employment, mode of delivery, and infant gender.

#### 2.3.2. SICS

The SICS (Self-efficacy in Infant Care Scale) was used to measure MSE. This 46-item scale has four dimensions of “developmental promotion, general health care, safety, and diet”. Each item refers to one parenting task, and women are supposed to assess their belief of confidence in performing various parenting tasks from “Not confident at all to do it” (0 point) to “Definitely confident I can do it” (100 point). The higher average score women acquire indicates the higher MSE level women have. The internal consistency of SICS was 0.96, and ranged between 0.86 and 0.96 for its four dimensions [31]. The test–retest reliability coefficient of SICS was 0.93. In the current study sample, the Cronbach’s alpha coefficient was 0.94 for the total scale.

#### 2.3.3. EPDS

The EPDS (Edinburgh Postnatal Depression Scale) is used for postpartum depression screening of first-time mothers. The instrument has a total score of 0–30 which includes ten items, and each item scores from 0 to 3 [32]. A lower score means a better mental health status of the women. The reported internal consistency of the Chinese version EPDS is 0.87; its concurrent validity was 0.79 with the BDI (Beck Depression Inventory) [33]. The Cronbach’s alpha coefficient of the EPDS was 0.85 in the present study. Total EPDS scores of 10 and 13 have been recommended as the threshold scores of mild and severe postnatal depression in mainland China, respectively [33].

#### 2.3.4. PSSS

The PSSS (Postnatal Social Support Scale) was used to measure Chinese women’s perception of received support after childbirth [34]. This 20-item tool is based on a four-point Likert-type scale, with each item being scored 0 (never), 1 (rarely), 2 (sometimes), or 3 (often). The total PSSS score ranges from 0 to 60 points. The higher score a mother achieves implies the more social support she receives. The reported internal consistency of this tool is 0.89 [34]. The test–retest reliability coefficient of PSSS was 0.92; its content validity was 0.90. In the present study, the Cronbach’s alpha coefficient was 0.90 for the total scale.

### 2.4. Data Analysis

Data were analyzed using SPSS Statistics 21.0. Descriptive statistics were used to describe the socio-demographic characteristics of floating women by means (SD), and frequencies (proportions). Paired sample *t*-tests were conducted to compare the scores of MSE, EPDS and social support at the two time points. Chi-squared tests were performed to compare the proportions of floating women with an EPDS score of 10 or above, and 13 or above from 6 weeks to 12 weeks postpartum, respectively.

## 3. Results

### 3.1. Participant Profile

In total, 674 socio-demographic questionnaires were collected; the participants’ profile is summarized in Table 2. The mean age of participants was 25.82 (3.38), and all were married. Of these, only 35.2% of women had a family income of RMB > 5000 (USD 700)/per month, person. Only 27.0% of respondents had a university degree, whereas 61.6% of them had an unskilled occupation.

### 3.2. MSE

#### 3.2.1. The Mean Scores of SICS

The mean scores of SICS were 67.16 (14.35) at 6 weeks postpartum and 68.71 (15.00) at 12 weeks postpartum. In its four dimensions of SICS, the scores of general health care were lowest, and the safety scores were highest (Table 3).

#### 3.2.2. Comparison of the Mean SICS Scores at 6 and 12 Weeks Postpartum

In the present study, 64.5% of participants (*n* = 435) completed both the 6-week and 12-week questionnaires. Thus, this subset was selected to explore the changing trends in MSE. The mean scores of SICS and its four dimensions consistently improved from 6 weeks postpartum to 12 weeks postpartum, and the increases in scores were statistically significant (*p* < 0.05) (Table 4).

### 3.3. Postpartum Depression

#### 3.3.1. Mean EPDS Scores 

The mean scores of the EPDS remained almost stable from 11.19 (4.89) at 6 weeks postpartum to 11.18 (5.34) at 12 weeks postpartum. However, the proportions of floating women with EPDS scores of 10 or above, and 13 or above, both decreased from 6 to 12 weeks postpartum (Table 5).

#### 3.3.2. Comparison of the Mean EPDS Scores at the Two Time Points

The reduction in the mean EPDS scores, as well as proportions of the 435 women with EPDS scores of 10 or above and 13 or above, were consistently statistically significant (*p* < 0.05), from comparisons between the two times points (Table 6).

### 3.4. Postnatal Social Support

#### 3.4.1. The Mean PSSS Scores

The mean PSSS score was 37.04 (10.15) at 6 weeks postpartum, and increased to 38.68 (10.46) at 12 weeks postpartum. In terms of scores in the four dimensions, the emotional support scores were highest; in contrast, the informational support scores were lowest, as illustrated in Table 7.

#### 3.4.2. Comparison of the Mean PSSS Scores at 6 and 12 Weeks Postpartum

The increase in the mean PSSS score among 435 participants had statistical significance from 6 to 12 weeks postpartum (*p* < 0.001). The mean scores of its four dimensions likewise exhibited a statistically significant improvement (*p* < 0.05) between 6 weeks and 12 weeks postpartum (Table 8).

## 4. Discussion

### 4.1. MSE

In the current research, the mean MSE scores of first-time mothers among rural-to-urban floating women were 67.16 (14.35) at 6 weeks postpartum and 68.71 (15.00) at 12 weeks postpartum. In contrast, the reported MSE scores of new mothers among city residents at the same time points were 74.92 (11.05) and 77.78 (11.13) [14,21]. The findings showed that these floating women had a lower level of MSE compared with the city-resident women. By comparing between the two time points, the mean scores of MSE and its four dimensions exhibited statistically significant increases from 6 to 12 weeks postpartum, which were consistent with the study results from city residents undertaken in China [14,29]. The findings from Porter and Hsu [35] likewise showed that the MSE scores of American women significantly increased between 4 weeks and 12 weeks postpartum. There are some reasons possibly accounting for the improvements in MSE with the passage of time. First of all, the increase in MSE levels in mothers’ perception may be related to an increase in parenting experience, as supported by Bandura’s theory [36]. First-time mothers were repeatedly engaged in the caregiving process as time went on; therefore, these parenting routines were possibly becoming more familiar to them. Moreover, this could be due to the fact that because infants’ social smiles increase fourfold from the second to the sixth month of life, this enabled mothers to become more sensitive and attached to their baby. With the positive changes in infants, combined with the development of mother–infant relationships, women might find it easier to understand and fulfill their infant’s needs and probably feel more successful and confident in their abilities of taking care of a baby during the first few months postpartum [37].

In terms of the four dimensions of the SICS, the lowest scores at the two time points were consistently related to the general health care of infants. For instance, scores on the item of “give first aid to my baby when there is an object blocking her/his throat or nostrils” was only 26.62, and item of “give proper care when my baby has a seizure” was 28.28; these values were lower than 30.00 points at 6 and 12 weeks postpartum. The items of “give mouth care to my baby every day”, “use a suction bulb correctly when my baby has phlegm”, “relieve my baby’s gas pain”, and “give proper care when my baby gets mild diarrhoea”, all scored lower than 50.00 at the two time points. These findings indicated that this population of floating women had lower confidence in parenting tasks surrounding baby health care, i.e., common baby disease management and emergency care. These parenting tasks associated with a low MSE score in the research implied an urgent need for alterations of the current parenting training. New parenting training from health professionals during mother’s hospitalization and postnatal home visiting should ensure to include parenting information about common baby disease management and emergency care to increase the relatively low MSE levels for rural-to-urban floating women.

### 4.2. Postpartum Depression

At 6 and 12 weeks postpartum, the mean scores of the EPDS were 11.19 (4.89) and 11.18 (5.34), much higher than the results from Chinese primiparous women among city residents, whose EPDS scores were 9.09 (4.33) and 8.63 (4.41) at the same time points [14]. Furthermore, the EPDS scores in the present study were also much higher than previous research findings from Western countries. For instance, 2659 women in the United Kingdom had mean EPDS scores of 6.80 (4.28) at six weeks postpartum [38]. Another study investigated 410 Irishwomen, and found their mean score on the EPDS to 7.20 (4.40) at six weeks postpartum [18]. In terms of EPDS threshold scores, the proportions of rural-to-urban floating women with EPDS scores of 10 or above and 13 or above were 54.4% and 50.6% at 6 weeks postpartum, respectively; the corresponding percentages were 40.1% and 35.4% at 12 weeks postpartum. In comparison, the proportions of city-resident women with EPDS scores of 10 or above and 13 or above were 47.4% and 21.4% at 6 weeks postpartum, respectively; these were 38.3% and 18.2% at 12 weeks postpartum, respectively [14].

These results clearly indicate that a higher proportion of rural-to-urban floating women are likely to have PPD symptoms than city-resident women. Unbalanced allocations of health resources may have led to mental health disparities between the floating women and the city-resident women [6,7]. For example, a national survey on 5372 rural-to-urban floating women found that many migrant women received no antenatal care during the three months of pregnancy and had an insufficient number of antenatal visits in their entire pregnancy [5].

Comparing results at 6 and 12 weeks postpartum, the decrements in mean scores of EPDS, as well as proportions with EPDS scores of 10 or above and 13 or above, were all statistically significant, which demonstrated that the depressive symptoms of floating women alleviated as the time progressed. This finding was consistent with previous studies [14,35] in which the researchers observed that PPD exhibited a peak incidence at about six to eight weeks postpartum, and its symptoms would diminish with the passage of time [39,40]. However, the decreases in EPDS score (0.77 point) suggest that having a clinical change in this study needs to be discussed. According to the standard by Matthey [41], at least the four points of change in EPDS scores represents a real clinical change in the individual; none of the floating women in this study exhibited a clinically reliable alleviation from 6 and 12 weeks postpartum. Therefore, as a higher-risk group of PPD, first-time mothers among floating women require effective and accessible mental health care after childbirth, such as early PPD screening and timely therapeutic methods.

### 4.3. Social Support

In the present research, the mean scores of social support were 37.04 (10.15) and 38.68 (10.46) at 6 and 12 weeks postpartum, lower than prior findings from city-resident women in China using the same measurement of PSSS, whose social support scores were 40.99 (SD = 9.31) and 43.00 (SD = 9.55) at the same time points [14]. This means that these floating women did receive less social support after childbirth than city women. Some factors may have contributed to this finding. Firstly, baby-raising is not only an important matter for Chinese mothers, but for the entire family. Approximately 80% of urban grandmothers were reported to assist new city-resident mothers with looking after their babies [42]. However, most floating women lacked parenting support from the babies’ grandparents, because the majority of their parents lived in relatively undeveloped rural areas, and rarely moved to the city to live together due to the economic burden. Moreover, there are unavoidable obstacles for floating women to access various supports, such as social welfare and healthcare benefits, because of the longstanding household registration system in China [1,9].

In comparison with emotional and material support, floating women received less informational and appraisal support in their perception. In contrast, other studies in Hong Kong [43] and Finland [44] identified that mothers received sufficient information and adequate evaluative support from health professionals. It has been argued that no single support provider will be appropriate in all cases [14]. For example, women’s family members and friends are beneficial when offering emotional support (i.e., confidence and love) and material support (i.e., time and money); health professionals could be expected to give floating women much more informational support and evaluative support, such as professional parenting advice and instructions [13,14].

The period between 6 and 12 weeks postpartum exhibited the statistically significant increase in the mean scores of social support and its four dimensions, which was consistent with the previous research [13,14]. This indicated that these floating women perceived they had received more social support with the passage of time. However, whether the increased scores on the self-report tool represented an important health effect needs to be explored in further research.

There are several limitations to the study. Although every eligible woman was approached during the ten-month recruitment period, the researchers did not record their overall basic socio-demographic features; therefore, undetected forms of sample bias could not be ruled out. Moreover, self-report tools were used to measure the main variables, which may have led to bias of social desirability due to the traditional belief of “domestic shame should not be made public”, especially concerning PPD [45]. Furthermore, due to the time and financial limitations, no further follow-up three months postpartum was conducted in the research. Thus, for the sake of better understanding the changing trends in parenting outcomes, mental health and social support among floating women, further studies with various quantitative and qualitative methods in longer-term surveillance could be conducted in different cities or regions in China.

In summary, primiparous women among rural-to-urban migrant population in this study had a low MSE level; they especially had lower confidence in parenting tasks of baby health care, such as common baby diseases management and emergency care. New parenting training by health professionals during women’s hospitalization and postnatal home visiting should ensure to include parenting information of common baby diseases management and emergency care to increase the relatively low MSE levels for floating women. In the present research, primiparous women among the floating population acquired a low level of postpartum social support. In comparison with emotional and material support, floating women perceived that they received less informational and appraisal support. Health professionals are strongly recommended to afford floating women much more informational support and appraisal support, i.e., professional parenting suggestions, guidelines and evaluation. From 6 to 12 weeks postpartum, these floating women experienced statistically significant improvements in MSE and social support. However, the increased MSE and social support scores indicate that clinical effects need to be investigated in future research. In this study, a large proportion of rural-to-urban floating women appeared to have PPD; the symptoms were alleviated from 6 to 12 weeks postpartum. However, reductions in the EPDS scores with the passage of time were too small to represent clinical significance.

## 5. Conclusions

China has the largest group of rural-to-urban floating women worldwide, most of whom are of childbearing age. However, few studies have focused on parenting outcomes for floating women; the changing trends in MSE and significant influencing factors of PPD and social support are not clear for these women. This research aimed to monitor the changing trends in MSE, PPD and postnatal social support for these women to fill this research gap.

The present research findings indicated that primiparous women among the rural-to-urban migrant population had a clearly negative status of parenting outcomes and mental health, as well as lack of social support after childbirth. Although these floating women experienced statistically significant increases in MSE and social support and decreases in PPD from 6 to 12 weeks postpartum, this reduction over time was too slight to represent significantly clinical effects. In future, tailored evidence-based interventions are greatly needed to promote floating women’s MSE, mental wellbeing and social support in the early stages of motherhood. As a higher-risk group for PPD, primiparous women among floating populations require effective and accessible mental health care after childbirth, such as early PPD screening and timely therapeutic methods.

## Figures and Tables

**Table 1:** Participants’ inclusion and exclusion criteria.

Inclusion Criteria	Exclusion Criteria
Rural-to-urban floating women;	Women with severe mental or physical illness; Infants with severe mental or physical illness.
≥18 years old;	
First-time mothers with a healthy baby;	
Being able to respond to the questionnaires in Chinese.	

**Table 2:** Participant profile (*n* = 674).

Variables	Mean (SD)	Frequency	Percentage (%)
Childbearing age	25.82 (3.38)		
Marital status			
Married		674	100
Divorced		0	0
Single		0	0
Monthly family income (RMB/Per person)			
<3000 (USD < 420)		151	22.4
3001–5000 (USD 420–700)		286	42.4
>5000 (USD > 700)		237	35.2
Education			
Middle school graduate or lower		145	21.5
High school graduate		347	51.5
University graduate or higher		182	27
Employment			
Professional		31	4.6
Unskilled		415	61.6
Skilled		49	7.3
Unemployed		179	26.6
Mode of childbirth			
Natural childbirth		417	61.4
Assisted childbirth		119	17.5
C-section		138	20.5
Baby gender			
Boy		392	58.2
Girl		282	41.8

**Table 3:** The mean scores of SICS and its four dimensions at 6 (*n* = 544) and 12 (*n* = 435) weeks postpartum.

Variable	Time Point	Mean (SD)	Minimum	Maximum
MSE score				
(0–100)	6 weeks	67.16 (14.35)	37.17	98.91
	12 weeks	68.71 (15.00)	37.39	99.57
Developmental Promotion				
(0–100)	6 weeks	73.32 (12.93)	32.67	100.00
	12 weeks	75.88 (12.72)	45.33	100.00
General Health Care				
(0–100)	6 weeks	49.19 (17.32)	10.67	98.67
	12 weeks	53.13 (20.06)	13.33	99.33
Safety				
(0–100)	6 weeks	84.26 (13.00)	51.67	100.00
	12 weeks	83.79 (13.53)	50.00	100.00
Diet				
(0–100)	6 weeks	74.70 (14.79)	38.80	100.00
	12 weeks	71.60 (19.30)	17.50	100.00

**Table 4:** Comparison of the mean scores of SICS and its four dimensions at 6 and 12 weeks postpartum (*n* = 435).

Variable	Time Point	Mean (SD)	Paired Differences Mean (95% CI)	*t* Value	*p* Value
MSE score					
(0–100)	6 weeks	65.28 (14. 90)	3.44 (2.79, 4.09)	10.362	<0.001
	12 weeks	68.71 (15.01)			
Developmental Promotion			4.28 (3.54, 5.02)	11.360	<0.001
	6 weeks	71.59 (13.44)			
	12 weeks	75.88 (12.72)			
General Health Care			6.24 (5.12, 7.35)	11.006	<0.001
	6 weeks	46.89 (20.98)			
	12 weeks	53.13 (20.06)			
Safety			1.20 (0.40, 2.01)	2.947	0.003
	6 weeks	82.58 (13.61)			
	12 weeks	83.78 (13.53)			
Diet			1.42 (0.58, 2.25)	3.321	0.001
	6 weeks	71.60 (19.30)			
	12 weeks	73.02 (15.53)			

**Table 5:** The mean EPDS scores and the proportions with EPDS scores of 10 or above and 13 or above at 6 (*n* = 544) and 12 weeks (*n* = 435) postpartum.

Variable	Time Point	Mean (SD)	Frequency	Percentage (%)
EPDS score				
	6 weeks	11.19 (4.89)		
	12 weeks	11.18 (5.34)		
EPDS scores of 10 or above				
	6 weeks		296	54.4
	12 weeks		220	50.6
EPDS scores of 13 or above				
	6 weeks		218	40.1
	12 weeks		154	35.4

**Table 6:** Comparison of the mean EPDS scores and proportions with EPDS scores of 10 or above and 13 or above at 6 and 12 weeks postpartum (*n* = 435).

EPDS	6 Weeks	12 Weeks	*t*/X^2^ Value	*p* Value
Mean (SD) ^a^	11. 95 (4.90)	11.18 (5.35)	5.536	<0.001
Threshold ^b^ N (%)				
				
≥10	269 (61.8)	220 (50.6)	^__^	<0.001
<10	166 (38.2)	215 (49.4)		
				
≥13	208 (47.8)	154 (35.4)	^__^	0.003
<13	227 (52.2)	281 (64.6)		

^a^ by paired sample *t*-test (*t* value); ^b^ by paired sample chi-squared test.

**Table 7:** The mean scores of PSSS and its four dimensions at 6 (*n* = 544) and 12 weeks postpartum (*n* = 435).

Variable	Time Point	Mean (SD)	Minimum	Maximum
PSSS score				
(0–60)	6 weeks	37.04 (10.15)	15	60
	12 weeks	38.68 (10.46)	11	60
Emotional Support				
(0–20)	6 weeks	10.62 (2.67)	4	15
	12 weeks	10.95 (2.61)	4	15
Material support				
(0–20)	6 weeks	10.44 (3.22)	2	15
	12 weeks	10.41 (3.02)	2	15
Informational support				
(0–20)	6 weeks	7.02 (3.10)	0	15
	12 weeks	7.79 (3.33)	2	15
Evaluation of support				
(0–20)	6 weeks	8.96 (2.98)	2	15
	12 weeks	9.53 (3.09)	2	15

**Table 8:** Comparison of PSSS score and scores of its four dimensions at 6 and 12 weeks postpartum (*n* = 435).

Variable	Time Point	Mean (SD)	Paired Differences Mean (95%CI)	*t* Value	*p* Value
PSSS score			2.72 (2.11, 3.33)	8.774	<0.001
	6 weeks	35.95 (10.36)			
	12 weeks	38.68 (10.46)			
Emotional Support			0.61 (0.39, 0.83)	5.426	<0.001
	6 weeks	10.34 (2.74)			
	12 weeks	10.95 (2.61)			
Material support			0.29 (0.06, 0.51)	2.501	0.013
	6 weeks	10.12 (3.28)			
	12 weeks	10.41 (3.02)			
Informational support			0.85 (0.59, 1.10)	6.565	<0.001
	6 weeks	6.94 (3.21)			
	12 weeks	7.79 (3.33)			
Evaluation of support			0.98 (0.77, 1.20)	8.980	<0.001
	6 weeks	8.55 (2.99)			
	12 weeks	9.53 (3.09)			

## Data Availability

The data presented in this study are available on request from the corresponding author. The data are not publicly available due to privacy restrictions.
